# The expression and clinical significance of syncytin-1 in serum exosomes of hepatocellular carcinoma patients

**DOI:** 10.1515/biol-2022-0930

**Published:** 2024-09-19

**Authors:** Xuewei Zhuang, Xiao Shi, Hui Zhao, Shuai Shang, Xinyu Xu, Xiaomin Wang, Xin Zheng, Jing He

**Affiliations:** The Third Provincial Hospital Affiliated to Shandong University, 250000, Jinan, Shandong, China; Tai’an Municipal Hospital, 271000, Tai’an, Shandong, China; Dezhou Hospital of Traditional Chinese, 250000, Dezhou, Shandong, China

**Keywords:** hepatocellular carcinoma, syncytin-1, exosome, alpha-fetoprotein, tumor marker

## Abstract

This study aimed to investigate the expression and clinical significance of syncytin-1 in the serum exosomes of hepatocellular carcinoma (HCC) patients. Serum samples were collected from 61 patients with newly diagnosed HCC and 61 healthy individuals. Exosomes were extracted from serum samples and identified using transmission electron microscopy and Western blot. The relative expression levels of syncytin-1 in exosomes were determined by real-time quantitative PCR. The protein expression levels of alpha-fetoprotein and syncytin-1 in HCC patients were detected using enzyme-linked immunosorbent assay (ELISA). Statistical analysis was performed to evaluate the sensitivity and specificity of serum exosomal syncytin-1 in diagnosing HCC. The relationships between syncytin-1 expression and clinical pathological features were analyzed using receiver operating characteristic curve analysis. The results showed that the expression level of syncytin-1 in the serum of patients with newly diagnosed HCC was significantly higher than that in the normal control group (*P* < 0.0001). Using pathological diagnosis as the gold standard, the sensitivity and specificity of syncytin-1 for the auxiliary diagnosis of HCC were 91.3% and 75.5%, respectively, which were significantly higher than those of alpha-fetoprotein *(P* < 0.0001). The relative expression level of serum exosomal syncytin-1 was significantly associated with lymph node metastasis, degree of differentiation, and CNLC staging of HCC patients *(P* < 0.05). In conclusion, syncytin-1 in serum exosomes has high sensitivity and specificity for diagnosing HCC and can serve as a novel tumor marker for early screening, detection, and staging of HCC.

## 1 Introduction

Liver cancer is a common malignant tumor of the digestive system in China. According to data released by GLOBOCAN 2020, the global incidence and mortality rates of liver cancer rank fifth and third, respectively, making it one of the deadliest malignancies [1]. Early stages of liver cancer often progress silently, and by the time it is clinically detected, it has usually reached an advanced stage, leading to low 5-year survival rates. It is estimated that 55% of global liver cancer patients are from China [2]. The causes of liver cancer include hepatitis B virus, hepatitis C virus infection, excessive alcohol consumption, non-alcoholic fatty liver disease, cirrhosis caused by other factors, and a family history of liver cancer [3,4,5]. Primary liver cancer can be histologically classified into hepatocellular carcinoma (HCC), intrahepatic cholangiocarcinoma, and a mixed type of HCC-intrahepatic cholangiocarcinoma. Although these three types are all primary liver cancers, they exhibit significant differences in etiology, treatment methods, and prognosis, with HCC being the most common pathological type [6,7].

Despite advancements in the diagnosis and treatment of HCC, the prognosis for patients in China remains unsatisfactory, with the overall 5-year net survival rate increasing only from 11.7% in 2000–2004 to 14.1% in 2010–2014 [8]. Patients usually exhibit no obvious symptoms in the early stages. By the time clinical symptoms appear, the disease has often progressed to the middle or late stages. Common clinical manifestations include liver pain, hepatomegaly, and ascites. At this point, most patients have missed the optimal window for surgery and have a poor prognosis [9]. Current diagnostic methods for HCC include imaging techniques, tumor markers, and tumor tissue biopsy. However, in clinical practice, there are cases where alpha-fetoprotein (AFP) is negative, and ultrasound, computed tomography, and magnetic resonance imaging results are atypical. It often occurs in the early stages of HCC, where a definitive diagnosis is usually difficult. Tissue biopsy is an invasive procedure and not easily widespread. Therefore, there is a need for a non-invasive, effective diagnostic method to provide a basis for the early diagnosis of HCC.

Liquid biopsy technology is a diagnostic technique that analyzes blood and body fluid samples from patients. Clinical trials have confirmed that liquid biopsy technology has high sensitivity and can be used for early tumor screening [10]. Additionally, liquid biopsy is minimally invasive, easy to obtain, repeatable, and capable of dynamic detection, providing more comprehensive clinical references and wide applicability. Exosomes are nano-sized extracellular vesicles (30–150 nm) endogenously produced by almost all cell types. They facilitate intercellular communication by delivering mRNA, non-coding RNA (i.e. microRNA and long non-coding RNA), and proteins to recipient cells. It has also been found that the secretion of exosomes by cancer cells is higher than that by normal cells [11].

Tumor markers play a crucial role in the early diagnosis of HCC. Common tumor markers include AFP, AFU, and DCP, but their sensitivity ranges from 67.7 to 81.7%, with specificities ranging from 70.7 to 90% [12]. The detection capability for large liver cancers is limited, and small liver cancers often go undetected. Neither individual nor combined detection methods achieve a sensitivity of 90%. Therefore, identifying new molecular diagnostic and therapeutic targets is essential for the early diagnosis and prognosis of HCC. Research has shown that the envelope protein syncytin-1 of the human endogenous retrovirus W1 is associated with conditions such as preeclampsia, malignant tumors of the reproductive system, lymphomas, and melanomas [13]. Syncytin-1 can activate various types of malignant tumors, including colorectal cancer, endometrial cancer, ovarian cancer, and testicular cancer [14,15,16]. However, there are few reports on the expression and significance of syncytin-1 in serum exosomes of HCC. This study aimed to detect the expression levels of syncytin-1 in the serum exosomes of HCC patients and analyze the correlation between syncytin-1 expression and clinical pathological parameters, thereby providing new insights into early clinical diagnosis of HCC.

## 2 Materials and methods

### 2.1 Clinical data

We collected serum samples from 61 patients diagnosed with primary HCC at Tai’an Municipal Hospital from July 2021 to July 2022. All patients diagnosed with primary HCC had not received any surgical, radiation, or chemotherapy treatments. Postoperative pathological results confirmed HCC in all cases. The exclusion criteria were as follows: ① presence of other malignant tumors; ② females who were pregnant or lactating; and ③ presence of abnormal cardiac and renal functions. Additionally, serum samples from 61 individuals undergoing health examinations at our hospital were collected as a healthy control group. This study was approved by the Ethics Committee of Tai’an Municipal Hospital, and all participants provided informed consent prior to enrollment.


**Informed consent:** Informed consent has been obtained from all individuals included in this study.
**Ethical approval:** The research related to human use has been complied with all the relevant national regulations, institutional policies and in accordance with the tenets of the Helsinki Declaration, and has been approved by the Ethics Committee of Tai’an Municipal Hospital.

### 2.2 Reagents

Hifair III 1st Strand cDNA Synthesis SuperMix for qPCR (gDNA digester plus) kit (Yeasen Biotechnology (Shanghai) Co., Ltd.), Hieff UNICON Universal Blue qPCR SYBR Green Master Mix kit (Yeasen Biotechnology (Shanghai) Co., Ltd.), anti-ALIX antibody 1:500 (HuaBio Company), anti-CD63 antibody 1:500 (HuaBio Company), anti-CD81 antibody 1:1,000 (HuaBio Company), anti-Calnexin antibody 1:1,000 (HuaBio Company), HRP conjugated goat anti-rabbit IgG antibody 1:50,000 (HuaBio Company).

### 2.3 Methods and result determination

#### 2.3.1 Extraction of serum exosomes

Pre-processing of Specimens: Serum samples were thawed at room temperature and transferred to centrifuge tubes. They were centrifuged at 300*g*, 4°C for 30 min. The supernatant was then transferred to new centrifuge tubes and centrifuged at 3,000*g* for 30 min. The remaining supernatant was further centrifuged at 20,000*g* for 30 min.

Ultracentrifugation method was used to extract serum exosomes. The supernatant was centrifuged with the Beckman Coulter Optima XPN-100 Intelligent Ultracentrifuge at 100,000*g* for 80 min, repeated twice. After discarding the supernatant, exosomes were obtained in approximately 100 μL volume. The exosomes were repeatedly pipetted for thorough mixing, transferred to RNase-free centrifuge tubes, and then stored at −80°C for future use.

#### 2.3.2 Exosome identification under electron microscope

Using the negative staining technique, 30 μL of the resuspended solution was placed on a dedicated copper grid for electron microscopy and left at room temperature for 2 min. After staining with 20 μL of 2% phosphotungstic acid for 10 min, exosomes were observed using a Japan Electron JEOL transmission electron microscope (JEM-1200EX II) with a magnification of 25,000×. Exosomes were shown as disc-shaped membrane-bound vesicles, with a diameter of 30–150 nm under transmission electron microscopy.

#### 2.3.3 Immunoblotting

Exosomes were obtained from 20 serum samples using ultracentrifugation. Then, exosomal total protein was extracted using an exosome protein extraction kit. SDS-PAGE electrophoresis was performed with the following steps: gel preparation, sample loading, electrophoresis, gel extraction, membrane transfer, blocking, primary antibody incubation, secondary antibody incubation, and visualization. The expression levels of surface-specific marker proteins CD63, Alix, and CD81 on exosomes were assessed, using protein markers to determine molecular weight. The negative control protein was Calnexin.

#### 2.3.4 Extraction of total RNA

Total RNA was extracted by adding TRIZOL lysis reagent to the exosome suspension (*n* = 61). After thorough mixing, the mixture was left at room temperature for 5 min. Subsequently, 200 μL of chloroform was added and mixed for 5 min, followed by a 5-minute incubation. The mixture was then centrifuged at 12,000*g* for 15 min at 4°C. The upper aqueous phase (approximately 500 μL) was carefully transferred to a new tube, and an equal volume of isopropanol was added. The mixture was inverted and allowed to stand for 10 min at room temperature. After centrifugation at 12,000*g* for 10 min at 4°C, the supernatant was discarded, and 500 μL of 75% ethanol was added to the pellets, followed by centrifugation at 7,500*g* for 5 min. The supernatant was discarded, and the pellet was air-dried before being resuspended in 40 μL of DEPC-treated water.

#### 2.3.5 Measurement of total RNA concentration and purity

The concentration and purity of total RNA were assessed by a NanoDrop 2000C UV-visible spectrophotometer (Thermo Evolution, USA). The OD260/OD280 values of the RNA samples were measured.

#### 2.3.6 Reverse transcription of RNA to cDNA

Reverse transcription of RNA was carried out using the Hifair III 1st Strand cDNA Synthesis SuperMix for qPCR (with gDNA digester plus) kit on ice. The reaction system was 20 μL, including 3 μL of DNA digester mix, 1 μg of extracted RNA, and DEPC-treated water to 15 μL. The reaction mixture was first incubated at 42°C for 3 min for digestion, followed by the addition of 5 μL of 4× Hifair III SuperMix Plus for reverse transcription. The reaction conditions were as follows: 25°C for 5 min, 55°C for 15 min, 85°C for 5 min, and then held at 4°C. The reverse-transcribed cDNA was stored at −80°C for future use.

#### 2.3.7 qRT-PCR detection of syncytin-1 mRNA

The Hieff UNICON Universal Blue qPCR SYBR Green Master Mix kit was used for the qRT-PCR reaction, and all operations were performed on ice. Each sample was run in triplicate. The reaction system was 20 μL, including 10 μL of PCR SYBR Green Master Mix, 0.4 μL of Forward Primer (10 μM), 0.4 μL of Reverse Primer (10 μM), 2 μL of cDNA, and 7.2 μL of ddH2O. The reaction conditions were as follows: initial denaturation at 95°C for 2 min, followed by 40 cycles of denaturation at 95°C for 10 s, annealing and extension at 60°C for 30 s. Dissociation curve analysis was performed by gradually increasing the temperature from 72 to 95°C. GAPDH was used as the internal reference gene.

syncytin-1 forward primer: 5′-GCAACTGCTATCACTCTGCCACT-3′,

syncytin-1 reverse primer: 5′-GAGTATGGGTACGGAGGGTTTC -3′

#### 2.3.8 Measurement of serum AFP levels

Serum AFP levels were measured using the electrochemiluminescence method with the Roche Cobas e602 electrochemiluminescence fully automated immunoassay system and original imported reagents. The measurements were conducted strictly according to the manufacturer’s instructions. The normal reference range for AFP is 0 to 7 ng/mL.

#### 2.3.9 Enzyme-linked immunosorbent assay (ELISA)

The ELISA method was employed to detect the expression levels of syncytin-1 protein in exosomes. The assay was conducted using a kit, and the empty well was used for blank calibration. The absorbance (OD value) of each well was measured sequentially at a wavelength of 450 nm.

### 2.4 Statistical analysis

All experiments were repeated three times, each performed in triplicate. IBM SPSS Statistics 27 and GraphPad Prism software were used for data analysis and visualization. The clinical experimental data in this study were calculated and analyzed using the 2^-ΔΔCT^ formula to obtain the relative expression level of syncytin-1. The data between groups did not conform to a normal distribution. Therefore, the Mann–Whitney *U*-test was used for comparisons between the two groups, and analysis of variance was employed for comparisons within groups. Receiver operating characteristic curves (ROC curves) were constructed based on the relative expression levels of syncytin-1 and serum AFP levels. The Youden index (sensitivity + specificity −1) was used to determine the cut-off value. A significance level of *P* < 0.05 was considered statistically significant for all tests.

## 3 Results

### 3.1 Identification of exosomes

**Figure 1:**
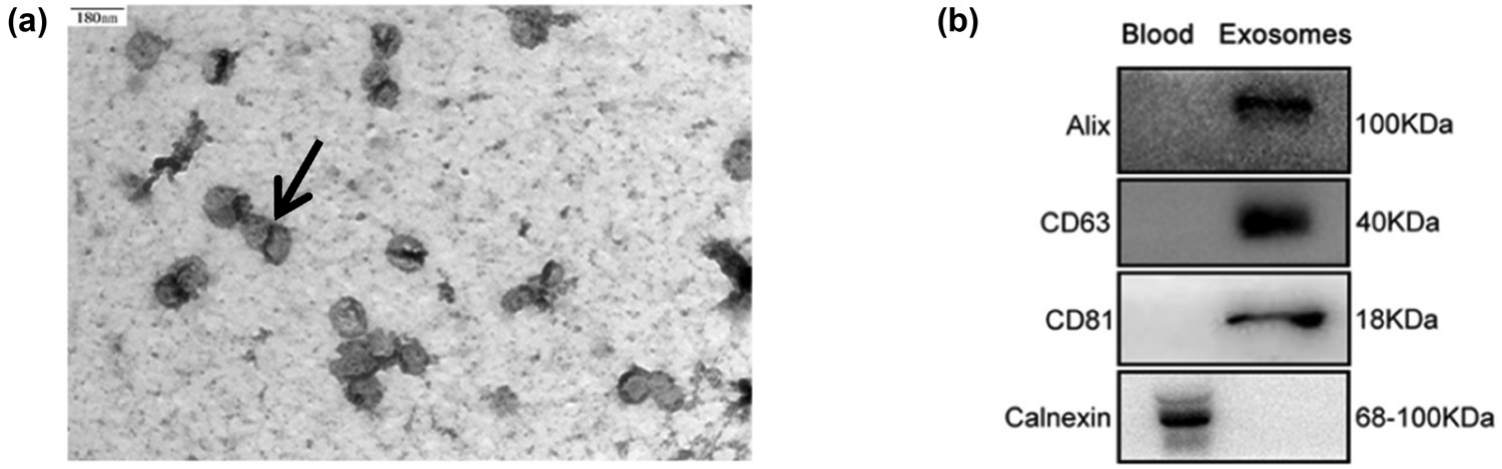
Identification of exosomes: (a) electron microscopy; (b) western blot analysis.

Electron microscopy was used to identify the extracted exosomes (Figure 1a). Under a transmission electron microscope, the exosomes exhibited a diameter ranging from 30 to 150 nm, displaying a characteristic of a double-disc structure. Western blot (Figure 1b) showed positive expression of exosomal surface markers Alix, CD63, and CD81 in the serum precipitate, while the negative marker Calnexin was not expressed.

### 3.2 Total RNA determination results

The optical density ratio of total RNA extracted from the serum of HCC patients ranged from 50 to 220 ng/μL, with OD260/OD280 values ranging from 1.8 to 2.1.

### 3.3 Relative expression levels of syncytin-1 in serum exosomes of HCC patients

**Figure 2:**
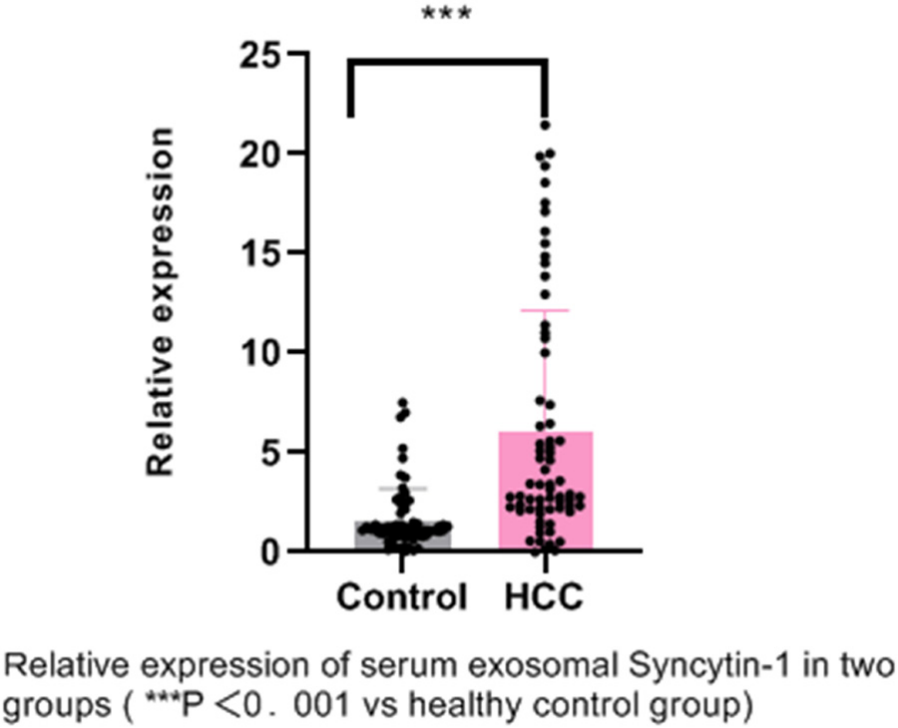
Serum mRNA levels of syncytin-1 measured by qRT-PCR.

There was no significant difference in age and gender between the HCC and control groups. Syncytin-1 in serum exosomes was expressed in both HCC patients and the healthy control group. However, the expression level of syncytin-1 in the HCC group was significantly higher than that in the healthy control group, with statistical significance (*P* < 0.001) (Figure 2).

### 3.4 The relationships between relative expression levels of syncytin-1 in serum exosomes and clinical pathological features of HCC patients

The relative expression levels of syncytin-1 in serum exosomes of HCC patients showed statistically significant differences in relation to lymph node metastasis, degree of differentiation, CNLC staging, and whether they were infected with hepatitis B virus (*P* < 0.05), but not with age, gender, or history of alcohol consumption *(P* > 0.05) (Table 1).

**Table 1:** The relationships between syncytin-1 expression in HCC patients and clinical pathological features

Pathological parameters	Case number	Relative syncytin-1 expression level	*P* value
**Age (years)**			0.0885
<60	28	7.243(0.102–21.47)	
≥60	33	4.474(0.02–17.56)	
**Gender (** ***n***)			0.3003
Male	40	5.164(0.018–19.4)	
Female	21	7.079(1.287–18.56)	
**Differentiation degree (** ***n***)			0.0075
Mid-high	41	11.08(1.534–9.4)	
Low	20	3.366(2.058–6.34)	
**CNLC staging (** ***n***)			0.0147
I	17	3.649(0.017–13.87)	
II	13	8.497(1.301–19.40)	
III	17	6.593(1.814–17.56)	
IV	14	3.653(0.156–12.95)	
**Distant metastasis (** ***n***)			0.0079
Yes	33	3.635(0.156–11.4)	
No	28	7.319(0.416–19.4)	
**History of alcohol consumption (** ***n***)			0.2622
Yes	32	5.459(0.1565–17.56)	
No	29	7.997(0.0178–20.03)	
**Infected with hepatitis B virus**			
Yes	38	7.453(0.1565–21.47)	0.0082
No	23	3.244(0.4159–10.02)	

### 3.5 ELISA examination

**Figure 3:**
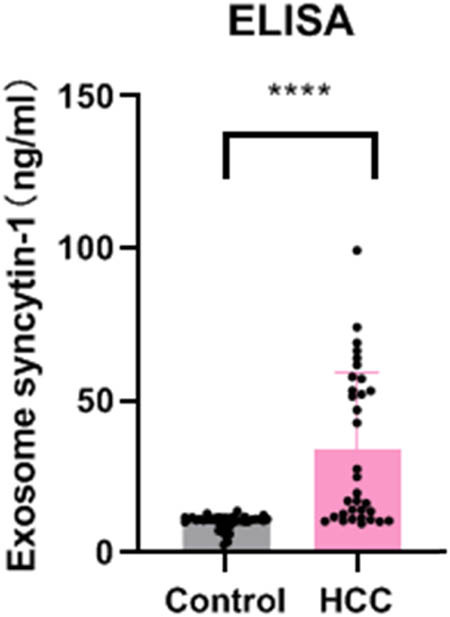
Serum protein levels of syncytin-1 measured by ELISA.

Results from ELISA examination showed that the expression level of syncytin-1 in serum exosomes was significantly higher in the HCC group compared to the control group (Figure 3).

### 3.6 The diagnostic efficiency of serum exosomal syncytin-1 in HCC patients

**Figure 4:**
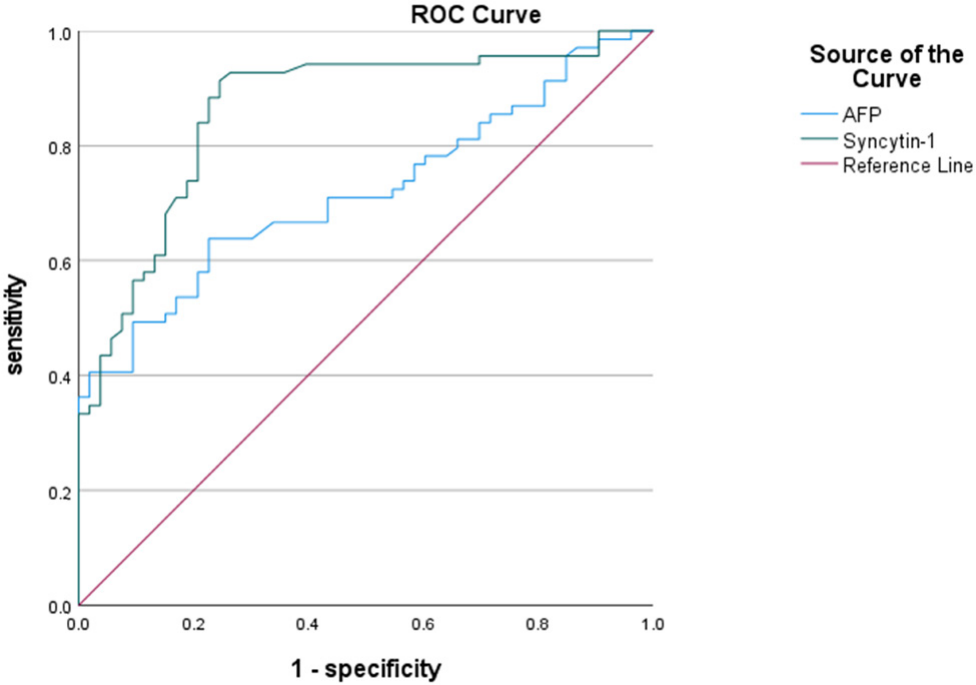
The ROC curves for serum exosomal syncytin-1 and AFP expression levels in the diagnosis of HCC.

Based on the relative expression levels of syncytin-1 in serum exosomes of HCC patients and healthy controls, we constructed an ROC curve. The cut-off value for syncytin-1 was 0.668, with an AUC of 0.863 (95% CI: 0.795–0.931, *P* < 0.0001). The sensitivity and specificity were 91.3% and 75.5%, respectively. Another ROC curve was constructed based on the serum AFP levels of HCC patients and healthy controls. The cut-off value for AFP was 0.406, with an AUC of 0.715 (95% CI: 0.625–0.806, *P* < 0.0001). The sensitivity and specificity were 63.2% and 77.4%, respectively. The AUCs from the two ROC curves were significantly different (differed by 0.148, *P* < 0.0001), suggesting that syncytin-1 has a better diagnostic value than AFP (Figure 4).

## 4 Discussion

Primary HCC is currently the fourth most common malignant tumor and the second leading cause of tumor-related deaths in China, posing a serious threat to the lives and health of our people [17]. Patients with HCC often have a concealed onset, high malignancy, and are prone to liver failure and death after surgery, which results in a high mortality rate [18,19]. Therefore, studying the early diagnosis of HCC is of great clinical significance.

Exosomes are extracellular vesicles with a double-layered membrane structure, ranging in size from 30 to 150 nm. They are widely present in various bodily fluids, including blood, urine, and saliva, and can be produced by most cells. Exosomes play diverse physiological roles, including immune regulation, cell differentiation and migration, and intercellular signaling [20,21,22]. Exosomes act as key messengers in the complex intercellular communication that occurs during cancer progression, as they can transmit information between tumor cells or between malignant and normal cells. Increasing evidence shows that specific cellular components originating from primary tumor cells accumulate in exosomes. These exosomes can then regulate functional responses through interactions with target tumor cells and by restructuring various types of cancer cells [23]. Research on exosomes primarily aims to explore their capacity as carriers of disease biomarkers. For instance, exosomes found in plasma and cerebrospinal fluid have been discovered to contain α-synuclein, a protein associated with Parkinson’s disease [24,25,26]. Previous study also demonstrates that exosomes isolated from urine can reflect acute kidney injury [27]. Furthermore, exosomes have been identified as carriers of markers for pancreatic cancer and lung cancer [28,29].

Syncytin-1, a member of the human endogenous retrovirus W gene family, is a ubiquitous envelope protein in the human reproductive system, localized in the syncytiotrophoblast layer during early pregnancy, playing a crucial role in fetal development [15,30]. Inoue et al. [16] observed cell proliferation phenomena during estrogen therapy for endometrial cancer. Further studies have shown that transforming growth factor-beta acts as a regulatory switch for the fusion and non-fusion activities of syncytin-1. In the absence of transforming growth factor-beta, syncytin-1 expression exhibits fusion activity; in its presence, it can increase tumor cell proliferation. Thus, non-fusion activity of syncytin-1 may lead to a malignant proliferation of tumors. In recent years, increasing evidence has confirmed abnormal expression of HERVW in various diseases, participating in the occurrence and development of diseases such as cancer, autoimmune diseases, and neurological disorders [17–19].

The results of this study demonstrate that serum exosomal syncytin-1 is expressed in both HCC patients and the healthy control group. However, the expression level of syncytin-1 in the HCC group is significantly higher than that in the healthy control group. Additionally, the relative expression level of serum exosomal syncytin-1 in HCC patients correlates significantly with lymph node metastasis, degree of differentiation, and CNLC staging. This suggests that a higher relative expression level of serum exosomal syncytin-1 may be associated with increased lymph node metastasis and later CNLC staging. Therefore, the relative expression level of serum exosomal syncytin-1 in HCC patients holds clinical diagnosis value.

Serum AFP is a commonly used tumor marker for HCC diagnosis and holds a certain reference value, but it exhibits poor sensitivity and specificity for detecting HCC. The results of this study revealed that the sensitivity and specificity of serum exosomal syncytin-1 for HCC were 91.3% and 75.5%, respectively, which were significantly higher than AFP, suggesting that the diagnostic efficacy of syncytin-1 is higher than that of AFP. This result provides a foundation for further research in the application of syncytin-1 in HCC diagnosis.

## 5 Strengths and limitations

This study represents the first analysis investigating the correlation between syncytin-1 expression in serum exosomes of HCC patients and clinical pathological parameters. By focusing on this novel biomarker in serum exosomes, the study provides valuable insights into the potential role of syncytin-1 in HCC diagnosis and prognosis. However, the limitations of this study should also be addressed. First, the study was conducted at a single center, which may limit the generalizability of the findings to broader populations. In addition, the sample size of the study was limited, which might affect the statistical power and reliability of the results. Lastly, subgroup analysis based on different patient characteristics or disease stages was not performed, which could provide more detailed insights into the correlation between syncytin-1 expression and specific clinical parameters. Future studies should involve larger sample sizes recruited from multiple centers to enhance the robustness and generalizability of the findings. Preclinical and animal studies are also warranted to elucidate the molecular mechanisms underlying the involvement of syncytin-1 in HCC development, which could provide insights into potential therapeutic targets or diagnostic markers for HCC [31].

## 6 Conclusion

In summary, serum exosomal syncytin-1 demonstrates higher sensitivity and specificity compared to AFP. It can serve as a more accurate, simple, and safe early screening biomarker. Therefore, syncytin-1 holds significant practical value in early HCC screening and can complement computed tomography scans for mutual confirmation, thereby achieving the goal of early detection of liver cancer.
